# Supplementary material for: Impact of introducing a standardized nutrition protocol on very premature infants’ growth and morbidity
Source: PLoS One. 2020 May 21;15(5):e0232659. doi: 10.1371/journal.pone.0232659 (PMC7241817; doi:10.1371/journal.pone.0232659)
Supplement: Attachment — Submitted filename: 200109 WITTWER et HASCOET Plos one highlight correction.doc [file pone.0232659.s006.doc]

**Impact of introducing a standardized nutrition protocol on very premature infants’ growth and morbidity**

Short title: Nutrition protocol and premature infants’ growth

**Wittwer Apolline*, Hascoët Jean-Michel***

* Department of Neonatology, Maternité Régionale, CHRU NANCY, DevAH University of Lorraine, France

Authors last name: Wittwer, Hascoët

Authors declare no conflict of interest.

Corresponding author: Wittwer; [a.wittwer@chru-nancy.fr](mailto:a.wittwer@chru-nancy.fr) , Tel: +33 3 83 34 29 34

Sources of Support: none

This retrospective study was registered by ClinicalTrials.gov under number NCT03217045, and by the CNIL (Commission Nationale de l’Informatique et des Libertés) under study number R2015-1 for the Maternity of the CHRU of Nancy.

ABREVIATIONS

BPD: bronchopulmonary dysplasia

BW: birth weight

CLD: chronic lung disease

CVL: central venous line

DGOS: Direction générale de l’offre de soins

ESPGHAN: European Society of Paediatric Gastroenterology, Hepatology and Nutrition Committee

GA: gestational age

HM: human milk

IUGR: intra uterine growth restriction

MOM: mother’s own milk

NEC: necrotizing enterocolitis

NICU: neonatal intensive unit care

PDA: patent ductus arteriosus

PGR: postnatal growth restriction

PMA: post menstrual age

SD: standard deviation

WGA: weeks of gestational age

**ABSTRACT**

Background: Inappropriate nutritional intake in premature infants may be responsible for postnatal growth restriction (PGR) and adverse long-term outcomes.

Objective: We evaluated the impact of an updated nutrition protocol on very premature infants’ longitudinal growth and morbidity, and secondly the compliance to this new protocol.

Design: All infants born between 26-32 weeks gestation (GA) were studied retrospectively during two 6-month periods before (group 1) and after (group 2) the introduction of an optimized nutrition protocol, in a longitudinal comparative analysis.

Results: 158 infants were included; 72 before and 86 after the introduction of the protocol (Group 1: (mean±SD) birthweight (BW) 1154±276 g, GA 29.0±1.4 weeks; Group 2: BW 1215±332 g, GA 28.9±1.7 weeks). We observed growth improvement in Group 2 more pronounced in males (weight z-score) at D42 (−1.688±0.758 vs. −1.370±0.762, p=0.045), D49 (−1.696±0.776 vs. −1.370±0.718, p=0.051), D56 (−1.748±0.855 vs. −1.392±0.737, p=0.072), D63 (−1.885±0.832 vs. −1.336±0.779 p=0.016), and D70 (−2.001±0.747 vs. −1.228±0.765 p=0.004). There was no difference in females or in morbidities between the groups. We observed low compliance to the protocol in both groups: similar energy intake but higher lipid intake in Group 1 and higher protein intake in Group 2.

Conclusion: The quality of nutritional care with a strictly-defined protocol may significantly improve weight gain for very preterm infants. As compliance remained low, an educational reinforcement is needed to prevent PGR.

**INTRODUCTION**

Inappropriate nutrition is an issue for premature infants as it may be responsible for postnatal growth restriction (PGR) and increased morbidity (1–4). *Embleton* (2) demonstrated that 55% of PGR (5) were related to a cumulative deficit in energy and protein intake. Malnutrition in the first weeks of life is associated with short stature and adverse outcomes in adulthood (6–8). The goal of nutrition is to allow body composition and outcomes similar to those of infants born at term (3,9). Optimizing nutrition needs to start from birth as the window for PGR prevention and catch-up growth is rather narrow (10). The prevention of nutrient deficits may be achieved through the implementation of optimized nutritional policies. More recent studies also demonstrated benefits on growth with the implementation of nutritional strategies (11–13).

We decided therefore to introduce a standardized nutritional protocol in our NICU and study outcomes on premature infants (including growth and morbidity).

In our unit, parenteral and enteral nutrition used to be adjusted according to the 2004 guidelines defining global nutritional intakes (14,15). From May 1^st^, 2014, we decided to update our policy according to more recent ESPGHAN and other recommendations (16–19) available at the time of the study, and to follow a strict nutritional protocol rather than only global guidelines.

The objective of this study was to evaluate the longitudinal impact of implementing this strictly-defined nutrition protocol on very premature infants’ growth and morbidity using a before/after comparison design, with a 6-months wash-out period. The secondary objective was to evaluate physicians’ compliance to the standardized protocol.

**Patients and Methods**

## **Study design**

All infants born at the maternity hospital and hospitalized in our NICU were studied retrospectively during two 6-month periods, separated by a 6-month washout period, from May 1 to October 31, 2013 (group 1) then from May 1 to October 31, 2014 (group 2), which occurred before and after the introduction of an optimized nutrition protocol. We performed a longitudinal comparative analysis between these two independent groups. Collection of data from the infants’ files was standardized.

**Methods**

This retrospective study was registered by ClinicalTrials.gov under number NCT03217045, and by the French ethic committee CNIL (“Commission Nationale de l’Informatique et des Libertés”) under study number R2015-1 for the Maternity of the CHRU of Nancy. Parents’ consent for using the collected clinical data of their infant was obtained and signed at admission.

Infants born between 26 to 32 weeks gestation (GA) and admitted to our NICU were included in the study. Infants who died before discharge or presented with any congenital malformation were excluded. The primary outcome measure was to evaluate the impact of introducing a well-defined nutrition protocol on the longitudinal growth of the infants up to the time of discharge. Weight was assessed by daily measurements, every morning, as defined in our routine policy of care. To account for variations in gestational and postnatal age, body weight was converted into a z-score using the Olsen preterm infants’ reference growth chart (20).

In order to account for the infants’ size at birth, we also analyzed each point z-score difference from birth z-score. PGR was defined by a weight z-score <-1,5 DS before 36 WOG or discharge (21).

Secondary outcomes were the incidence of necrotizing enterocolitis (NEC) as defined by Bell (22); interruption of nutrition and its duration; duration of parenteral nutrition; incidence of late onset sepsis; patent ductus arteriosus (PDA) requiring treatment; bronchopulmonary dysplasia (BPD) defined as the use of supplemental oxygen at 28 days of life; and chronic lung disease (CLD) defined as the need for supplemental oxygen at 36 weeks PMA. Antenatal steroids rate, ibuprofen, caffeine, and doxapram treatment were also recorded in the infants’ files.

Finally, we evaluated weekly actual nutritional intake of the infants fed according to the guidelines compared to the new protocol to define the compliance of our team to the 2 policies. Enteral and parenteral nutrition were modified according to ESPGHAN recommendations. Proteins were prescribed at a higher rate in group 2, with a onset at birth, of 1.5 vs. 0.5 g/kg/d, and a goal of 3.5 vs. 2.5 g/kg/d. Lipids were prescribed earlier in group 2 and at higher rate of 1 g/kg/d on day 1 vs. 0.5 g/kg/d starting on day 2, with a goal of 3.5 vs. 3 g/kg/d. Enteral nutrition was initiated from day 1 in both groups but with an increase of 15-20 in group 2 vs.10-15 ml/kg/d in group 1 (Table 1).

Use of mother’s own milk was promoted, but when it was not available banked donor’s milk was used. Milk fortification was initiated from 80 ml/kg/d in both groups.

The parenteral solutions were prepared by the hospital pharmacy according to medical prescription.

In order to evaluate compliance to the protocol, energy, protein, lipid, carbohydrate, and sodium intake were reported weekly for each patient after collection of the raw data in the patient’s individual daily file. Human milk content was assumed to be an average of 70 kcal/100 mL with 1.2 g protein, 4.2 g fat, and 7 g/100 mL carbohydrate(23) . Milk fortification and preterm infant formulas were also used and their formula compositions and nutritional content were based on the product labels.

Each intake (protein, carbohydrate, lipid) was compared to the theoretical recommendations of each nutritional protocol in use for the period. When the intake was out of the range of the recommendations, it was considered as not compliant to the protocol for the period.

Compliance to guidelines for each intake in the two groups was calculated by the average of compliant prescriptions compared with recommendations for each nutritional protocol in use for the period (in percentage +/- standard deviation).

## **Statistics**

Normally distributed data, assessed by a Shapiro-Wilk test of normality, are presented as mean values with SD; non-normally distributed data are presented as medians with the range of values. To evaluate differences between groups, we used the Student t test for continuous variables and the Chi^2^ test or Fisher exact test when appropriate for categorical variables. For continuous variables not normally distributed, we used the Mann-Whitney U test. Categorical data are presented as actual numbers or percentages. Postnatal evolution of growth variables was assessed longitudinally by z-score. Observed differences were considered statistically significant if *P* < 0.05. All analyses were performed with SYSTAT 12 software (2007, Systat Software Inc®, San Jose CA, USA).

# RESULTS

## **Demographic and clinical characteristics of the population**

From May to October 2013, 85 eligible infants were included in the study. Thirteen were excluded: twelve died, and one file was lost. The remaining 72 infants (Group 1) had a mean birthweight (BW) of 1154 ± 276 g, and a mean GA of 29.0 ± 1.4 weeks. From May to October 2014, 90 eligible infants were included in the study. Four were excluded: two died and two files contained missing data. The remaining 86 infants (Group 2) had a mean BW of 1215 ± 332 g and a mean GA of 28.9 ± 1.7 weeks.

The clinical characteristics of the study population are displayed in Table 2. The groups were similar, with no significant differences for sex, birth weight, GA, prenatal steroids, PDA, or BPD. We observed a significant difference for PDA requiring surgery and for the duration of treatment with doxapram which did not impact the results (Table 2).

## **Primary outcome: impact on longitudinal raw weight and weight z-score**

Postnatal weight loss occurred in both groups during the first week of life, with no difference in the minimal global weight on day 7 (1121±233 g in Group 1 vs. 1159±305 g in Group 2; p = 0.385). Mean weight loss was 2.8% in Group 1 and 4.9% in Group 2 (NS). There was no significant difference in raw weight between the two groups from birth to day 77. However, when looking at weight z-scores, we observed significant differences between the 2 periods from day 42 up to day 70 (Figure 1). Of note, z-score decrease was significantly more important (about 0.5 z-score) and delayed in Group 1 as compared to Group 2 (day 70 vs. day 21, respectively) (Figure 1).

The analysis of each point z-score difference from birth was above -1 at each time point, ranging from -0.775 to -0.981, without significant difference between groups or sex.

Looking at weight z-score evolution with regards to gender, we observed a significant difference between male and female infants; there was a significant difference in males from day 42 to day 70 (Figure 2A), but not in females (Figure 2B).

**Secondary outcomes**

We evaluated the potential impact of our new standardized protocol on population morbidity (Table 2). We evaluated the tolerance of enteral nutrition because of a faster increase in enteral nutrition volume, but found no difference between the two groups for the rate of NEC.

While our new protocol allowed for faster achievement of full enteral nutrition, there was no significant difference in the duration of hospitalization, central venous line and parenteral nutrition duration (which corresponds with full enteral feeding), or the rates of infection (Table 2).

**Compliance to guidelines or standardized protocol**

We observed low compliance to the protocol in both groups with regards to protein, lipid, and caloric intakes with a high variability. Figure 3 presents the nutrition data up to D42. There were no further differences between the groups from D42 onwards.

Concerning caloric intakes, there was low compliance in both group throughout the observation period, but no significant differences in the median caloric intake between the 2 groups (Figure 3A). Compliance for caloric intake was 27.9%+/-19.8 in group 1 and 23%+/- 26.1% in group 2. Of note, Group 2 received a significantly higher protein intake and Group 1 received a significantly higher lipid intake from day 0 to day 42. The median protein intake varied from 1.2 to 3.1 g/kg/d at day 42 in Group 1 and from 1.5 to 3.5 g/kg/d at day 42 in Group 2, with significant differences between the two groups from day 7 to day 42 (Figures 3B and 3C). Compliance to guidelines was also low for protein intakes (53% +/-35.2 in group 1 and 38.6% +/- 26.4 in group 2), and for lipid intakes (26.9 % +/- 25.6 in group 1 and 36.6% +/- 31 in group 2).

# DISCUSSION

In this study, we showed that the introduction of a strict nutritional protocol, designed according to the most recent recommendations available at the time of the study, led to a higher weight gain velocity from the sixth to the eleventh week of life with a limitation of PGR in very preterm infants. We observed a significantly attenuated drop in weight z-score compared with infants receiving nutrition based on the older recommendations. The difference was not statistically significant after 40 weeks PMA because the number of infants discharged from the hospital increased, thus the analysis lacked power. Our results also showed that the minimal z-score was higher and occurred earlier in Group 2 than in Group 1, with a catch-up effect after day 35 not observed in Group 1. These results suggest that a strictly implemented nutritional protocol may indeed improve growth in very preterm infants, limiting PGR severity before discharge. Of note, there was no difference in raw weight between the two groups, confirming that raw weight is not a good indicator for following infants’ growth in daily practice. Indeed, weight z-score is a better predictor to diagnose growth restriction and adjust nutrition (24) .

There was a significant growth improvement between the two periods for males only. This difference observed in growth trajectory is consistent with Christmann V and Hack long term observations (21, 22). However, this difference may also be due in part to the fact that males had lower weight z-scores at birth than females in our population.

Early nutritional deficits were not regained before hospital discharge in our study. This could be explained by poor compliance to the protocol. A significant part of PGR in preterm infants has been linked to nutritional deficit, which is mainly caused by fear of metabolic intolerance or NEC (2,3). Looking at individual infant’s files to verify their actual nutrition intake with regards to guidelines (Group 1) or the strict protocol (Group 2), we observed low compliance in both groups for the first month of life and throughout the observation period. This is consistent with Lapillonne et al (27). Of note, we observed a significantly higher protein intake in Group 2, as expected when using the new protocol, but a significantly higher lipid intake in Group 1, with no differences in caloric intake between both groups. Maybe this low compliance was due to higher expectations, so despite higher intakes, compliance was not reached.

Thus, our two groups were demographically and clinically comparable, but their nutritional intake was qualitatively different. This point raises the question about the quality of growth in formerly premature infants (28). We can speculate that more protein intake is better because growth velocity is increased in our study. Previously published studies confirm that earlier and higher protein intake by preterm infants may increase weight gain velocity. Also they may achieve a leaner mass closer to that of full term infants (24). We need to improve our practice and strictly follow the nutritional protocol. Early growth improvement is very important because there seems to be a window, in the first weeks of life, when interventions may have an impact (29,30). We showed that despite appropriate energy intake and more protein intake, there is a catch-up growth but delayed by about one month. We speculate that protein intake may have a delayed maturation effect due to cumulative deficit during the first month (2).

When accounting for the size of the infants at birth, delta z-scores were stable with time and always above -1. This suggests that a better nutrition with more appropriate protein intake would prevent growth failure aggravation (31) related to nutrition, mainly observed in boys.

We evaluated the effects of our new protocol on clinical outcomes other than growth and did not find any difference except a higher rate of surgery for PDA in Group 2, and longer doxapram treatment in Group 2. These differences may be explained by a better availability of pediatrics surgeons from 2014, and by a modification in our protocol for doxapram treatment between the two periods. The rate of NEC was lower in group 2 but not statistically different despite that enteral nutrition was increased faster using the new protocol. This is consistent with good tolerance of early “aggressive” nutrition for preterm infants (2).

The two groups were similar for the duration of central venous line and parenteral nutrition. We expected a shorter duration with the new protocol because enteral feeding was increased faster and the central venous line should be removed earlier than before (140 ml/kg/day of enteral feeding after *versus* 160 ml/kg/day before). This part of the protocol was obviously not fulfilled. These results demonstrate that when a new protocol is introduced in a unit, medical caregivers must be trained and that a time for adaptation is needed, as a recent study suggested it (32).

The strengths of the study include the extensive retrospective standardized collection of data as well as the routinely measured weight values throughout the study period.

Conversely, our study has limitations. It was not a randomized trial; however, it would not be ethical to voluntarily limit nutritional support in some infants. Confounding factors, such as medical treatment and mechanical ventilation could have affected the results, but were not investigated in this cohort study. However, we may speculate that a 6-month washout period was short enough to prevent significant modifications in our practice and allow for a comparison of both cohorts. Other limitations include the retrospective collection of nutritional data and a high dropout rate due to hospital discharge. Thus, we cannot exclude that the lack of difference observed from D70 onwards may be related to a lack of power. There is also variability in the protein and the fat composition of human milk that we were not able to determine at the time of the study (33).

# CONCLUSION

Feeding preterm neonates is extremely challenging. Optimal nutrition with a low incidence of complications requires a well-organized structure. Hospital staff must acknowledge that preterm birth is also a nutritional emergency which could result in serious short- and long-term detrimental effects. Our data suggest that the quality of nutritional care using a strictly-defined protocol may improve weight gain for very preterm infants. The results are promising since this policy was able to limit PGR at discharge.

This study was based on an internal audit and we noted that what we think we are doing is not always what we are really doing. That kind of evaluation may be recommended to all units to point out what improvements can be made for optimizing nutritional care for these vulnerable infants.

Author contribution statement:

This study was designed by JM.Hascoët (JMH) and A.Wittwer (AW), directed and coordinated by JMH who provided conceptual and technical guidance for all aspects of the project.

AW collected the data and JMH analyzed it.

The manuscript was written by AW and commented by JMH.

REFERENCES

1.	Cooke R. Postnatal Growth in Preterm Infants: Have We Got it Right? J Perinatol. 2005;25(S2):S12‑4.

2.	Embleton NE, Pang N, Cooke RJ. Postnatal malnutrition and growth retardation: an inevitable consequence of current recommendations in preterm infants? Pediatrics. févr 2001;107(2):270‑3.

3.	Grover A, Khashu M, Mukherjee A, Kairamkonda V. Iatrogenic malnutrition in neonatal intensive care units: urgent need to modify practice. JPEN J Parenter Enteral Nutr. avr 2008;32(2):140‑4.

4.	Senterre T, Rigo J. Reduction in postnatal cumulative nutritional deficit and improvement of growth in extremely preterm infants: Nutritional deficit and growth in preterm infants. Acta Paediatr. févr 2012;101(2):e64‑70.

5.	Lee PA, Chernausek SD, Hokken-Koelega ACS, Czernichow P. International Small for Gestational Age Advisory Board Consensus Development Conference Statement: Management of Short Children Born Small for Gestational Age, April 24–October 1, 2001. Pediatrics. 6 janv 2003;111(6):1253‑61.

6.	Barker DJ. Intrauterine programming of coronary heart disease and stroke. Acta Paediatr Oslo Nor 1992 Suppl. nov 1997;423:178‑82; discussion 183.

7.	Smart JL, Dobbing J, Adlard BP, Lynch A, Sands J. Vulnerability of developing brain: relative effects of growth restriction during the fetal and suckling periods on behavior and brain composition of adult rats. J Nutr. sept 1973;103(9):1327‑38.

8.	Ehrenkranz RA, Dusick AM, Vohr BR, Wright LL, Wrage LA, Poole WK. Growth in the neonatal intensive care unit influences neurodevelopmental and growth outcomes of extremely low birth weight infants. Pediatrics. avr 2006;117(4):1253‑61.

9.	Monset-Couchard M, de Bethmann O, Kastler B. Mid- and long-term outcome of 166 premature infants weighing less than 1,000 g at birth, all small for gestational age. Biol Neonate. 2002;81(4):244‑54.

10.	Dusick AM, Poindexter BB, Ehrenkranz RA, Lemons JA. Growth failure in the preterm infant: can we catch up? Semin Perinatol. août 2003;27(4):302‑10.

11.	Andrews ET, Ashton JJ, Pearson F, Beattie RM, Johnson MJ. Early postnatal growth failure in preterm infants is not inevitable. Arch Dis Child - Fetal Neonatal Ed. mai 2019;104(3):F235‑41.

12.	Roggero P, Giannì ML, Orsi A, Amato O, Piemontese P, Liotto N, et al. Implementation of Nutritional Strategies Decreases Postnatal Growth Restriction in Preterm Infants. Denning PW, éditeur. PLoS ONE. 5 déc 2012;7(12):e51166.

13.	Rochow N, Fusch G, Mühlinghaus A, Niesytto C, Straube S, Utzig N, et al. A nutritional program to improve outcome of very low birth weight infants. Clin Nutr Edinb Scotl. févr 2012;31(1):124‑31.

14.	Nutrition and feeding of preterm infants. Committee on Nutrition of the Preterm Infant, European Society of Paediatric Gastroenterology and Nutrition. Acta Paediatr Scand Suppl. 1987;336:1‑14.

15.	Klein CJ. Nutrient requirements for preterm infant formulas. J Nutr. juin 2002;132(6 Suppl 1):1395S-577S.

16.	Agostoni C, Buonocore G, Carnielli VP, De Curtis M, Darmaun D, Decsi T, et al. Enteral nutrient supply for preterm infants: commentary from the European Society of Paediatric Gastroenterology, Hepatology and Nutrition Committee on Nutrition. J Pediatr Gastroenterol Nutr. janv 2010;50(1):85‑91.

17.	Lapillonne A, Razafimahefa H, Rigourd V, Granier M, others. La nutrition du prématuré. Arch Pédiatrie. 2011;18(3):313–323.

18.	Senterre T, Rigo J. [Parenteral nutrition in premature infants: practical aspects to optimize postnatal growth and development]. Arch Pédiatrie Organe Off Sociéte Fr Pédiatrie. sept 2013;20(9):986‑93.

19.	Senterre T, Rigo J. Optimizing early nutritional support based on recent recommendations in VLBW infants and postnatal growth restriction. J Pediatr Gastroenterol Nutr. nov 2011;53(5):536‑42.

20.	Olsen IE, Groveman SA, Lawson ML, Clark RH, Zemel BS. New intrauterine growth curves based on United States data. Pediatrics. févr 2010;125(2):e214-224.

21.	Zozaya C, Díaz C, Saenz de Pipaón M. How Should We Define Postnatal Growth Restriction in Preterm Infants? Neonatology. 2018;114(2):177‑80.

22.	Bell MJ, Ternberg JL, Feigin RD, Keating JP, Marshall R, Barton L, et al. Neonatal necrotizing enterocolitis. Therapeutic decisions based upon clinical staging. Ann Surg. janv 1978;187(1):1‑7.

23.	Billard H, Simon L, Desnots E, Sochard A, Boscher C, Riaublanc A, et al. Calibration Adjustment of the Mid-infrared Analyzer for an Accurate Determination of the Macronutrient Composition of Human Milk. J Hum Lact Off J Int Lact Consult Assoc. août 2016;32(3):NP19-27.

24.	Tremblay G, Boudreau C, Bélanger S, St-Onge O, Pronovost E, Simonyan D, et al. Body Composition in Very Preterm Infants: Role of Neonatal Characteristics and Nutrition in Achieving Growth Similar to Term Infants. Neonatology. 2017;111(3):214‑21.

25.	Christmann V, Roeleveld N, Visser R, Janssen AJWM, Reuser JJCM, van Goudoever JB, et al. The early postnatal nutritional intake of preterm infants affected neurodevelopmental outcomes differently in boys and girls at 24 months. Acta Paediatr Oslo Nor 1992. févr 2017;106(2):242‑9.

26.	Hack M, Schluchter M, Margevicius S, Andreias L, Taylor HG, Cuttler L. Trajectory and correlates of growth of extremely-low-birth-weight adolescents. Pediatr Res. févr 2014;75(2):358‑66.

27.	Iacobelli S, Viaud M, Lapillonne A, Robillard P-Y, Gouyon J-B, Bonsante F, et al. Nutrition practice, compliance to guidelines and postnatal growth in moderately premature babies: the NUTRIQUAL French survey. BMC Pediatr. 3 sept 2015;15:110.

28.	Thomas EL, Parkinson JR, Hyde MJ, Yap IKS, Holmes E, Doré CJ, et al. Aberrant adiposity and ectopic lipid deposition characterize the adult phenotype of the preterm infant. Pediatr Res. nov 2011;70(5):507‑12.

29.	Sudo N, Sawamura S, Tanaka K, Aiba Y, Kubo C, Koga Y. The requirement of intestinal bacterial flora for the development of an IgE production system fully susceptible to oral tolerance induction. J Immunol Baltim Md 1950. 15 août 1997;159(4):1739‑45.

30.	Maas C, Poets CF, Franz AR. Avoiding postnatal undernutrition of VLBW infants during neonatal intensive care: evidence and personal view in the absence of evidence. Arch Dis Child Fetal Neonatal Ed. janv 2015;100(1):F76-81.

31.	Martin CR, Brown YF, Ehrenkranz RA, O’Shea TM, Allred EN, Belfort MB, et al. Nutritional practices and growth velocity in the first month of life in extremely premature infants. Pediatrics. août 2009;124(2):649‑57.

32.	Stefanescu BM, Gillam-Krakauer M, Stefanescu AR, Markham M, Kosinski JL. Very low birth weight infant care: adherence to a new nutrition protocol improves growth outcomes and reduces infectious risk. Early Hum Dev. mars 2016;94:25‑30.

33.	Hascoët J-M, Skweres S, Marchal L, Chauvin M, Pierret C, Franck P. Impact of maternal nutrition and perinatal factors on breast milk composition after premature delivery. 2015.

Figures and table legends:

Table 1: Old guidelines compared to new protocol

Table 2: Clinical characteristics and outcomes of the study population

Figure 1: Longitudinal weight z-score evolution from birth to day 77. D=day, *p <0.05

Figure 2: A: Longitudinal weight z-score evolution from birth to day 77 in males. D=day, *p <0.05.

B: Longitudinal weight z-score evolution from birth to day 84 in females. D=day, p > 0.05 for all values.

Figure 3: Median and variability (IQR) for (A) energy, (B) protein, and (C) lipid intakes from birth to day 42. D=day, *p <0.05
